# A neuromusculoskeletal model to simulate the isokinetic ankle dorsiflexion test of spasticity

**DOI:** 10.1186/1757-1146-7-S1-A87

**Published:** 2014-04-08

**Authors:** Ruoli Wang, Örjan Ekeberg, Anders Fagergren, Johan Gäverth, Hans Forssberg

**Affiliations:** 1 Department of Computational Biology, KTH Royal Institute of Technology, Stockholm, Sweden; 2 AggeroMedtech AB, Stockholm, Sweden; 3 Department of Women's and Children's Health, Karolinska Institutet, Stockholm, Sweden

## Introduction

Spasticity is a motor disorder characterized by a velocity-dependent increase in tonic stretch reflexes [1], commonly seen in many neurological disorders. Clinically, spasticity is measured by an examiner rotating a joint and simultaneously estimating the resistance according to an ordinal scale. However, the limited reliability of the measurement and the impossibility to discriminate between the underlying neural (stretch reflex) and non-neural (i.e. muscle mechanics) contributions have been the motivation to develop methods describing resistance joint torque quantitatively. The aim of this preliminary study is to develop a forward neuromusculoskeletal model consisting of the explicit musculotendon, muscle spindle, and motoneuron pool, which can simulate the passive isokinetic ankle dorsiflexion test of spasticity.

## Material and methods

In the model, the plantarflexors were considered as a lumped representation of all the muscles. Dorsiflexors were not included in the model. The musculoskeletal geometry was based on the anthropometrical data from a healthy female (height: 1.62m, weight: 53kg). The hill-type musculotendon model was used to simulate the musculotendon dynamics of the lumped plantarflexors. Activation dynamics were modeled as a first order differential equation. The hybrid v^0.6^ model was used to model the firing characteristics of the muscle spindle [2]. The input-output relation of the α-motoneuron pool can be simplified as a sigmoid function. The contributions of the moment from the passive muscle properties and the stretch reflex to the total resistance torque were computed from 0° to 40° ankle dorsiflexion at two constant angular velocities (5°s^-1^ vs. 236°s^-1^).

## Results and discussions

**Figure 1:**
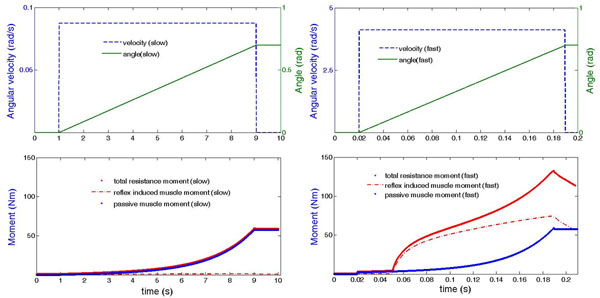
The contributions of the moment from passive muscle properties and the stretch reflex to the total resistance moment when *α*-motoneuron pool properties were specified. The angular velocities and the position of the ankle joint were prescribed in the simulation.

Compared to the fast ankle rotation, there was almost no stretch reflex-induced moment in the slow ankle rotation (Figure 1), which agrees to the definition of the spasticity. It indicates that the current neuromusculoskeletal model can describe the individual contributions to the total resistance moment. In the future, by comparing the experimental measurements and the predicted moment, the important spasticity related parameters e.g. α-motoneuron pool properties may be identified individually.
